# Early Treatment with Intranasal Neostigmine Reduces Mortality in a Mouse Model of *Naja naja* (Indian Cobra) Envenomation

**DOI:** 10.1155/2014/131835

**Published:** 2014-05-14

**Authors:** Matthew R. Lewin, Stephen P. Samuel, David S. Wexler, Philip Bickler, Sakthivel Vaiyapuri, Brett D. Mensh

**Affiliations:** ^1^Center for Exploration and Travel Health, California Academy of Sciences, 55 Music Concourse Drive, San Francisco, CA 94118, USA; ^2^Department of Clinical Medicine, Trinity College Dublin, Dublin 2, Ireland; ^3^Department of Chemistry and Biochemistry, University of California, Berkeley, Berkeley, CA 94920, USA; ^4^Department of Anesthesia and Perioperative Care, 505 Parnassus Avenue, University of California, San Francisco, CA 94122, USA; ^5^Institute for Cardiovascular and Metabolic Research, School of Biological Sciences, University of Reading, Reading, UK; ^6^Janelia Farm Research Campus, Howard Hughes Medical Institute, Ashburn, VA, USA

## Abstract

*Objective*. Most snakebite deaths occur prior to hospital arrival; yet inexpensive, effective, and easy to administer out-of-hospital treatments do not exist. Acetylcholinesterase inhibitors can be therapeutic in neurotoxic envenomations when administered intravenously, but nasally delivered drugs could facilitate prehospital therapy for these patients. We tested the feasibility of this idea in experimentally envenomed mice. *Methods*. Mice received intraperitoneal injections of *Naja naja* venom 2.5 to 10 times the estimated LD50 and then received 5 **μ**L neostigmine (0.5 mg/mL) or 5 **μ**L normal saline by nasal administration. Animals were observed up to 12 hours and survivors were euthanized. *Results*. 100% of control mice died. Untreated mice injected with 2.5× LD50 *Naja naja* died at average 193 minutes after injection, while 10 of 15 (67%) of treated mice survived and were behaviorally normal by 6 hours (*P* < 0.02). In the 5× LD50 group, survival was prolonged from 45 minutes to 196 minutes (*P* = 0.01) and for 10× LD50 mice, survival increased from 30 to 175 minutes (*P* < 0.02). *Conclusion*. This pilot suggests that intranasal drugs can improve survival and is the first direct demonstration that such an approach is plausible, suggesting means by which treatment could be initiated before reaching the hospital. Further investigation of this approach to neurotoxic and other types of envenomation is warranted.

## 1. Introduction

Bites from venomous snakes kill more people in the developing world than some of the world's better recognized and better studied neglected tropical diseases [1]. Recent estimates suggest that worldwide there are more than 5 million snakebites occurring each year. Though not all bites are by venomous snakes or result in poisoning, up to 2 million of these bites result in the injection of venom, with hundreds of thousands of significant injuries and as many as 94,000 to 125,000 deaths occurring primarily in India, Southeast Asia, and sub-Saharan Africa [2, 3]. The vast majority of snakebites occur in impoverished, rural populations with limited access to medical treatment. Mortality from snakebite is unequivocally linked to socioeconomic markers of poverty and even a successful hospital treatment can cause economic ruin. A recent study from the Indian state Tamil Nadu analyzed how patients hospitalized for snakebite paid for their expenses: 40% took loans, 20% sold stored crops, 15% sold valuables 10% sold cattle, and many reported removing their children from school—all while incurring up to 12 years income worth of debt [2]. It is estimated that around 10,000 people die from snakebite each year in Tamil Nadu, alone [2], more than twice the total number of deaths claimed by landmines each year, worldwide [4, 5].

Neostigmine is an acetylcholinesterase inhibitor (AChEI) that is administered intravenously and is currently recommended by the WHO for the treatment of neurotoxic snakebite. Acetylcholinesterase inhibiting drugs such as neostigmine and edrophonium are thought to reduce the neuromuscular block from neurotoxic snakebite by increasing the amount of acetylcholine at the neuromuscular junction as it does in the treatment of myasthenia gravis or the reversal of nondepolarizing neuromuscular blocking agents [6–9]. Atropine or glycopyrrolate, in intravenous (IV) form, are usually coadministered with AChEIs to blunt the undesirable muscarinic effects of AChEIs. However, coadministration is not necessary in some clinical studies, for example, in myasthenia gravis patients receiving intranasal (IN) administration for up to one year [10, 11]. Interestingly, IN neostigmine has been used to treat myasthenia gravis in several studies [10–13] and we recently showed in a human study that it could reverse mivacurium-induced neuromuscular blockade by this route [14].

The present study tested the hypothesis that neostigmine, given IN, would be an effective initial treatment of* Naja naja* envenomed mice. The early use of AChEIs leads to a considerable increase in the LD50 in mice and rats having undergone experimental envenomation [15, 16]. Our study is distinguished from those by the replacement of parenteral neostigmine with topically applied IN neostigmine. The rationale for this study is that since neurotoxic snakebites often occur far from hospitals, by eliminating the need for injection (e.g., of parenteral neostigmine or intravenous antivenin), we may be able to shorten time to treatment and save lives.

## 2. Materials and Methods

### 2.1. Institutional

The study was approved by the animal research committee of a contract research laboratory in Hyderabad, India, an IACUC-certified laboratory and performed by a trained technician, a full-time DVM and one of us (MRL) who performed experiments at the facility.

### 2.2. Materials and Animals

Unfractionated* N. naja* venom was purchased from Sigma-Aldrich (St. Louis, MO, USA); neostigmine and atropine were purchased from Besse Medical (Ann Arbor, MI, USA). Venom and drugs were reconstituted in sterile water. Mice had access to water and food at all times. Polyvalent antivenom (Vins Bioproducts, Andhra Pradesh, India) was available at all times in the event of accidental envenoming of staff.

### 2.3. Methods

A small pilot study was carried out to assess the potency of the reconstituted lyophilized* N. naja* venom to test if it was comparable to published reports of other commercially available unfractionated, frozen, or lyophilized* N. naja* venom at 0.3 mg/kg [15, 17–19].

Mice were pseudorandomized in batches of 5 with tails marked 1 to 5 stripes by Sharpie felt tip pen to receive intraperitoneal (IP) injections of* N. naja* venom (2.5× LD50, *N* = 20; 5× LD50, *N* = 10 and 10× LD50, *N* = 10) concomitantly with atropine, which blunts the muscarinic effects of neostigmine and has previously been shown to have no effect on LD50 when experimentally injected with snake venom [16]. The IP agents (venom and atropine) were adjusted for the weight of each individual mouse by the facility veterinarian and injected by a single technician who was not aware of the hypothesis and who also recorded the survival times. Animals received either 5 *μ*L of 0.5 mg/mL neostigmine or 5 *μ*L of saline by IN administration by MRL. Animals in the 2.5× LD50 group received treatment or control 10 minutes after venom injection. In the 5× LD50 and 10× LD50 groups, animals received IN neostigmine 1-2 minutes after venom injection. Preliminary studies the mice were already severely disabled by 10 minutes after experimental envenomation with the higher doses of venom and neostigmine did not appear to help. Animals were observed continuously for up to 12 hours and assessed for signs of toxicity including respiratory distress, loss of spontaneous locomotor activity with the only endpoints being time to death or recovery. Dead mice were removed immediately and tail-band number was recorded on a data sheet reflecting the mouse's lot and individual band number as well as weight. Surviving animals were euthanized after 12 hours by the same technician who performed the experimental envenomation procedure. The technician, however, was blinded to knowing which mice had been treated with IN neostigmine or saline control.

### 2.4. Data Analysis and Presentation

Data were analyzed using GraphPad Prism (La Jolla, CA) and the *P* values presented in the figures were as calculated by nonparametric Mann-Whitney test. Envenomed mice were further characterized using a survival analysis that included censoring to account for the study being terminated at 12 hours (720 minutes) after dosing. To plot survival time on a single *y*-axis, the survival time data was normalized within each envenomation dose to the mean survival time of each control group and then multiplied by 100.

## 3. Results

**Figure 1:**
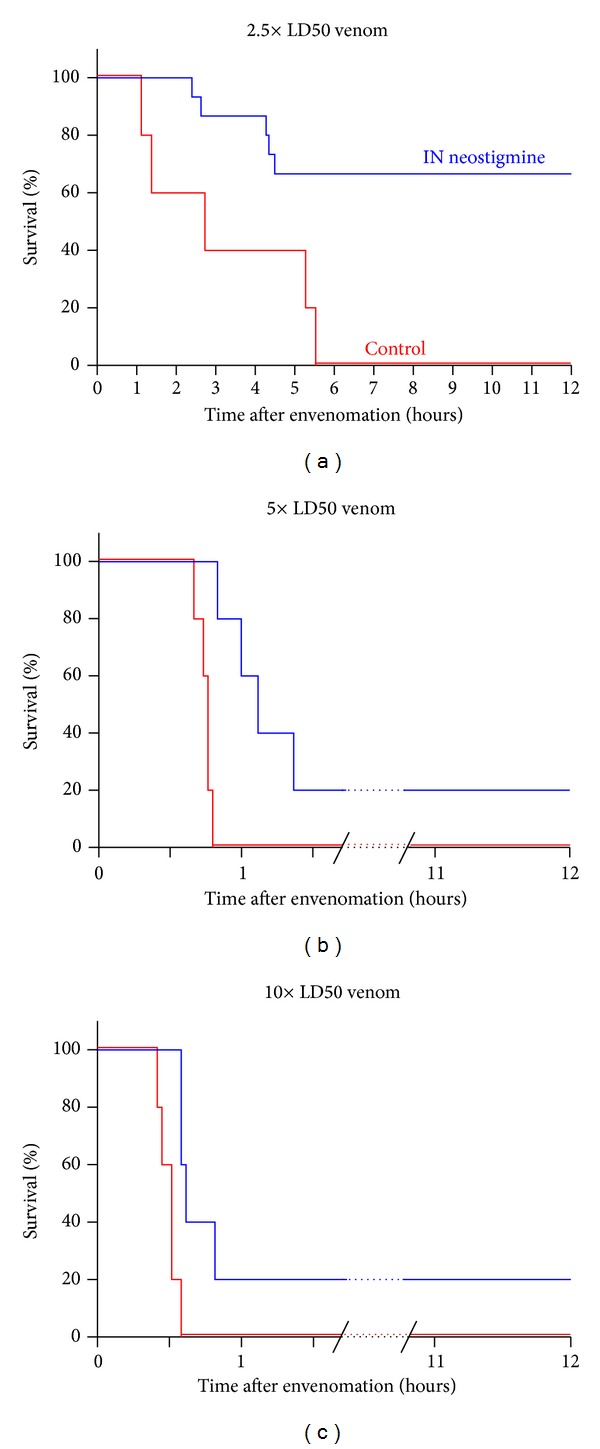
Kaplan-Meier plot of survival times in mice given 2.5 (a), 5 (b), or 10 (c) times the LD50 of* N. naja* venom and either a single dose of IN neostigmine (treatment groups, blue lines) or IN saline (control groups, red lines). *N* = 5 animals for each group, except *N* = 15 for the 2.5× LD50 treatment group. There were no significant differences in the mean weight of animals across groups.

Because snakebites in the community can result in a highly variable amount of venom being delivered to the patient, we sought to determine whether IN neostigmine could be effective in improving survival at several dosages of venom in our mouse model. There were no statistically significant differences in animal weight between any of the groups. Figures 1(a)–1(c) show the effects of neostigmine in mice envenomed with* Naja naja* venom at various concentrations: 2.5× LD50 (a), 5× LD50 (b), and 10× LD50 (c). As described above, the rationale for using IN neostigmine is to improve survival time from the moment of the snakebite. These results support our idea that early IN AChEI therapy could improve survival even after a potentially severe neurotoxic envenomation. Higher venom dosages resulted in earlier deaths, as expected, but for all dosages of venom, neostigmine provided a substantial and persistent window of increased survival.  Table 1 summarizes the data from all groups. At 2.5× LD50, envenomed mice died at an average of 193 minutes compared to 553 minutes (*P* < 0.02) for the treatment group (10/15 were euthanized after the arbitrary cutoff of 6 hours, but were behaving completely normally). At the 5× LD50 venom dosage, survival was prolonged from a mean of 45 minutes in the control group to 196 minutes in the treatment group (*P* = 0.01). Likewise, at the 10× LD50 venom dosage, mean survival was prolonged from 30 to 175 minutes (*P* < 0.02). Findings reached statistical significance even after reanalysis excluding surviving outliers in the 5× LD50 and 10× LD50 groups.

## 4. Limitations

Most bites in humans are on the extremities, but we chose the IP route for consistency and to replicate elements of previously published mouse studies [15–17]. Due to limitations of funding, we only tried one type of experimental envenomation using a curare-like snake neurotoxin; it is likely that the effects of IN AChEIs will vary across different venom types. Venoms contain a multitude of toxic peptides and proteins and published LD50 ranges vary widely between cobra species, subspecies and route of delivery (e.g., subcutaneous, intravenous, or IP) [15, 17–20]. Mice and humans differ greatly in their sensitivity to the same drugs [21, 22], and only one set of neostigmine to atropine concentrations was used. Thus, as with all transitions from preclinical to clinical usage, dosages will need to be optimized for human use. Fortunately, the development of IN neostigmine for the treatment of myasthenia gravis [10–13, 23, 24] provides a substantial head start for this transition. Only a single dose of IN neostigmine was administered, so it is not clear if mice would have survived longer with multiple treatments and no other AChEIs were tested. The concentration of neostigmine was significantly lower than that has been used in human studies, though total dose was comparable and the drugs were not aerosolized but dropped on the nares [10–14]. In the present study, atropine was coadministered with IN neostigmine through IP route. In previous mouse studies atropine has been administered IP without changing the LD50 of cobra venom [15]. We anticipate that an anticholinergic agent such as atropine (which can be administered IN) would potentially be administered with neostigmine to blunt untoward muscarinic effects of an AchEI should these effects be present with IN AChEI formulations in a human study [25]. Interestingly, in the year-long study by Sghirlanzoni and colleagues, patients self-administering IN neostigmine did not report any complications from IN neostigmine [11]. Broggini and colleagues tested the bioavailability of high dose IN neostigmine compared to IV administration in healthy human adults without coadministration of atropine and did not note any serious adverse events [23]. The IP-absorption kinetics of atropine are more reliable and proven than the IN absorption kinetics of atropine. Thus, if we had coadministered atropine IN instead of IP, we would be faced with the confoundedness that perhaps differential survival depended on intersubject differences in atropine-absorption kinetics. This confoundedness would be impossible to disambiguate from our central hypothesis in such a small study. By contrast, if survival depended on differential IN absorption kinetics (of neostigmine), that simply serves to further support the central hypothesis that neostigmine is the critical variable, especially in light of Gieu's results showing that IP atropine did not materially alter survival from experimental IP cobra envenomation [15]. We did not do necropsy on the mice. Similarly, we did not attempt any skin hemorrhagic or myonecrosis activity assays, though no unusual bleeding was noted. Mice were only observed for neurological manifestations of envenomation such as convulsion, hind limb paralysis, and respiratory distress after injection of reconstituted venom [17].

## 5. Discussion

Muscle-contraction-dependent respiration is a necessary condition for life amongst all mammals and virtually all vertebrates. The present finding builds on our earlier demonstration that IN neostigmine could reverse paralysis in an awake, experimentally paralyzed human subject [14]. To our knowledge, this is the first demonstration that a topically applied drug could reverse venom-induced neurotoxicity. We previously showed that nasal neostigmine could reverse mivacurium-induced paralysis in an awake human [14]. Together, these data provide proof-of-principle that venom-induced toxicity should be treatable in the out-of-hospital setting and provide early, life-saving interventions at low cost. There is evidence for neostigmine-resistant envenomings [26] in humans. Anil and colleagues showed that the mean time interval between bite and arrival to hospital was 4.5 h by which point the venom would have been entrenched at presynaptic axons [26]. Rapid death from krait bite most often comes as a result of the alpha-toxin and diaphragmatic paralysis and airway obstruction could be delayed by early AChEI therapy, but to our knowledge this idea has never been tested. In mouse studies, Guieu showed that among the drugs they tested only AChEIs consistently resulted in increases in* Naja* venom LD50s while atropine had no effect on the LD50 [15]. Similarly, Flachsenberger [16] showed that at otherwise lethal doses, all animals survived as a result of early AChEI treatment following IP administration of adder (*Acanthophis antarcticus*) venom. Flaschenberger further found that the expected survival time of animals subjected to even higher experimental venom doses was significantly extended. These animal [15, 16] and human morbidity and mortality studies suggest that if AChEIs can be administered during the initial, critical stage after envenomation there could be a survival benefit to human victims [16, 27–32].

Surprisingly, both the efficacy and optimal uses of antivenom and AChEI therapies for neurotoxic snakebite remain unproven even after decades of widespread use [2, 14, 33–38]. It has been argued that the development of more diverse and regionally specific antivenoms is the most cost effective means of combatting morbidity and mortality from snakebite in the developing world [39–41]. We argue that investment in repurposed, low-molecular-weight pharmaceuticals would be more cost effective in the long term because of their ease of use, heat stability, and safety profiles. IN administration of neostigmine has the potential to provide snakebite victims with significantly increased access to an effective treatment for neurotoxic snakebite while suggesting a strategy for the development of topically administered antidotes to hemotoxic, cardiotoxic, and other complex envenomation in the future. This type of innovation would save lives while significantly lowering the economic burden on individuals, families, communities, and governments. To date, no prospective human study has been done to analyze the effect of immediate AChEI administration in the setting of neurotoxic snakebite. The primary aim of this pilot study was to test if early IN administration of AChEIs and in principal any venom-inhibiting agent is plausible. The results of this study suggest that this is the case and that significant further study of this and other strategies is warranted.

## Figures and Tables

**Table 1:** Survival times for all venom dosages compared with and without IN neostigmine treatment. *P* values shown were as calculated by nonparametric Mann-Whitney test.

Treatment	Mean survival time in minutes (range)	*P* value	Number of mice per group
2.5× LD50 venom	193 (67–332)	<0.02	5
2.5× LD50 + neostigmine	553 (144–720)		15
5× LD50	45 (40–48)	=0.01	5
5× LD50 + neostigmine	196 (50–720)		5
10× LD50	30 (25–35)	<0.02	5
10× LD50 + neostigmine	175 (35–720)		5

## References

[1] Harrison RA, Hargreaves A, Wagstaff SC, Faragher B, Lalloo DG (2009). Snake envenoming: a disease of poverty. *PLoS Neglected Tropical Diseases*.

[2] Vaiyapuri S, Vaiyapuri R, Ashokan R (2013). Snakebite and its socio-economic impact on the rural population of Tamil Nadu, India. *PLoS ONE*.

[3] Chippaux J-P (2012). Epidemiology of snakebites in Europe: a systematic review of the literature. *Toxicon*.

[4] Wisborg T, Murad MK, Edvardsen O, Brinchmann BS (2008). Life or death. The social impact of paramedics and first responders in landmine-infested villages in northern Iraq. *Rural and Remote Health*.

[5] International Campaign to Ban Landmines (2014). *Dramatic Drop in Landmine CAsualties, Lives Saved as Clearance and Funding Reach New Peaks*.

[6] Walker MB (1935). Case showing the effect of prostigmin on myasthenia gravis. *Proceedings of the Royal Society of Medicine*.

[7] Banerjee RN, Sahni AL, Chacko KA, Vijay K (1972). Neostigmine in the treatment of Elapidae bites. *The Journal of the Association of Physicians of India*.

[8] Murphy GS, Szokol JW, Vender JS, Marymont JH, Avram MJ (2002). The use of neuromuscular blocking drugs in adult cardiac surgery: results of a national postal survey. *Anesthesia and Analgesia*.

[9] Mehndiratta MM, Pandey S, Kuntzer T (2011). Acetylcholinesterase inhibitor treatment for myasthenia gravis. *Cochrane Database of Systematic Reviews*.

[10] Ricciardi R, Rossi B, Nicora M, Sghirlanzoni A, Muratorio A (1991). Acute treatment of myasthenia gravis with intranasal neostigmine: clinical and electromyographic evaluation. *Journal of Neurology Neurosurgery and Psychiatry*.

[11] Sghirlanzoni A, Pareyson D, Benvenuti C (1992). Efficacy of intranasal administration of neostigmine in myasthenic patients. *Journal of Neurology*.

[12] Fossati A, Vimercati MG, Bandi GL, Formenti A (1990). Pharmacokinetic study of neostigmine after intranasal and intravenous administration in the guinea pig. *Drugs under Experimental and Clinical Research*.

[13] Di Costanzo A, Toriello A, Mannara C, Benvenuti C, Tedeschi G (1993). Intranasal versus intravenous neostigmine in myasthenia gravis: assessment by computer analysis of saccadic eye movements. *Clinical Neuropharmacology*.

[14] Lewin P MB, Heier T, Feiner J, Montauk L, Mensh BD (2013). Reversal of experimental paralysis in a human by intranasal neostigmine aerosol suggests a novel approach to the early treatment of neurotoxic envenomation. *Clinical Case Reports*.

[15] Guieu R, Rosso J-P, Rochat H (1994). Anticholinesterases and experimental envenomation by *Naja*. *Comparative Biochemistry and Physiology C: Pharmacology Toxicology and Endocrinology*.

[16] Flachsenberger W, Mirtschin P (1994). Anticholinesterases as antidotes to envenomation of rats by the death adder (*Acanthophis antarcticus*). *Toxicon*.

[17] Mukherjee AK, Maity CR (2002). Biochemical composition, lethality and pathophysiology of venom from two cobras—*Naja naja* and *N. kaouthia*. *Comparative Biochemistry and Physiology B: Biochemistry and Molecular Biology*.

[18] Binh DV, Thanh TT, Chi PV (2010). Proteomic characterization of the thermostable toxins from *Naja naja* venom. *Journal of Venomous Animals and Toxins Including Tropical Diseases*.

[19] Sakthivel G, Dey A, Nongalleima Kh (2013). *In vitro* and *in vivo* evaluation of polyherbal formulation against Russell's viper and Cobra venom and screening of bioactive components by docking studies. *Evidence-Based Complementary and Alternative Medicine*.

[20] Mohapatra B, Warrell DA, Suraweera W (2011). Snakebite mortality in India: a nationally representative mortality survey. *PLoS Neglected Tropical Diseases*.

[21] Michel AD, Thompson KM, Simon J, Boyfield I, Fonfria E, Humphrey PPA (2006). Species and response dependent differences in the effects of MAPK inhibitors on P2X7 receptor function. *British Journal of Pharmacology*.

[22] Geerts H (2009). Of mice and men: bridging the translational disconnect in cns drug discovery. *CNS Drugs*.

[23] Broggini M, Benvenuti C, Botta V, Fossati A, Valenti M (1991). Bioavailability of intranasal neostigmine: comparison with intravenous route. *Methods and Findings in Experimental and Clinical Pharmacology*.

[24] Ghigo E, Procopio M, Bellone J (1991). Intranasal administration of neostigmine potentiates both intravenous and intranasal growth hormone (GH)-releasing hormone-induced GH release in short children. *Journal of Clinical Endocrinology and Metabolism*.

[25] Rajpal S, Mittal G, Sachdeva R (2009). Development of atropine sulphate nasal drops and its pharmacokinetic and safety evaluation in healthy human volunteers. *Environmental Toxicology and Pharmacology*.

[26] Anil A, Singh S, Bhalla A, Sharma N, Agarwal R, Simpson ID (2010). Role of neostigmine and polyvalent antivenom in Indian common krait (*Bungarus caeruleus*) bite. *Journal of Infection and Public Health*.

[27] Kalantri S, Singh A, Joshi R (2006). Clinical predictors of in-hospital mortality in patients with snake bite: a retrospective study from a rural hospital in central India. *Tropical Medicine and International Health*.

[28] Yadavannavar MC, Patil AN (2013). A study of morbidity and subsidence of symptoms: subject to time gap of snakebite and treatment. *Journal of the Indian Medical Association*.

[29] Rao CP, Shivappa P, Mothi VR (2013). Fatal snake bites—sociodemography, latency pattern of injuries. *Journal of Occupational Medicine and Toxicology*.

[30] Halesha L BH, Lokesh AJ, Channaveerappa PK, Venkatesh K (2013). A study on the clinico-epidemiological profile and the outcome of snake bite victims in a tertiary care centre in southern India. *Journal of Clinical and Diagnostic Research*.

[31] Saravu K, Somavarapu V, Shastry AB, Kumar R (2012). Clinical profile, species-specific severity grading, and outcome determinants of snake envenomation: an Indian tertiary care hospital-based prospective study. *Indian Journal of Critical Care Medicine*.

[32] David S, Matathia S, Christopher S (2012). Mortality predictors of snake bite envenomation in southern India–a ten-year retrospective audit of 533 patients. *Journal of Medical Toxicology*.

[33] Ranawaka UK, Lalloo DG, de Silva HJ (2013). Neurotoxicity in snakebite-the limits of our knowledge. *PLoS Neglected Tropical Diseases*.

[34] Johnston CI, 'Leary MA O, Brown SG (2012). Death adder envenoming causes neurotoxicity not reversed by antivenom–Australian Snakebite Project (ASP-16). *PLoS Neglected Tropical Diseases*.

[35] Stone SF, Isbister GK, Shahmy S (2013). Immune response to snake envenoming and treatment with antivenom; complement activation, cytokine production and mast cell degranulation. *PLoS Neglected Tropical Diseases*.

[36] Ko JH, Chung WH (2013). Serum sickness. *The Lancet*.

[37] da Silva IM, Tavares AM (2012). Comparative evaluation of adverse effects in the use of powder trivalent antivenom and liquid antivenoms in Bothrops snake bites. *Revista da Sociedade Brasileira de Medicina Tropical*.

[38] Hasan SMK, Basher A, Molla AA, Sultana NK, Faiz MA (2012). The impact of snake bite on household economy in Bangladesh. *Tropical Doctor*.

[39] Warrell DA, Gutierrez JM, Calvete JJ, Williams D (2013). New approaches & technologies of venomics to meet the challenge of human envenoming by snakebites in India. *The Indian Journal of Medical Research*.

[40] Kini RM, Fox JW (2013). Milestones and future prospects in snake venom research. *Toxicon*.

[41] Keyler DE, Gawarammana I, Gutierrez JM (2013). Antivenom for snakebite envenoming in Sri Lanka: the need for geographically specific antivenom and improved efficacy. *Toxicon*.

